# Shoulder Traction as a Possible Risk Factor for C5 Palsy in Anterior Cervical Surgery: A Cadaveric Study

**DOI:** 10.3390/medicina60091429

**Published:** 2024-09-01

**Authors:** Ja-Yeong Yoon, Sung-Min Kim, Seong-Hwan Moon, Hak-Sun Kim, Kyung-Soo Suk, Si-Young Park, Ji-Won Kwon, Byung-Ho Lee

**Affiliations:** 1 Department of Orthopaedic Surgery, Daejeon Sun Hospital, Daejeon 34811, Republic of Korea; coffeesound2@gmail.com; 2 Department of Orthopaedic Surgery, College of Medicine, Kyung-Hee University Hospital at Gangdong, Seoul 05278, Republic of Korea; osdrksm83@gmail.com; 3 Department of Orthopedic Surgery, Yonsei University College of Medicine, Seoul 03722, Republic of Korea; shmoon@yuhs.ac (S.-H.M.); haksunkim@yuhs.ac (H.-S.K.); sks111@yuhs.ac (K.-S.S.); drspine90@yuhs.ac (S.-Y.P.); kwonjjang@yuhs.ac (J.-W.K.)

**Keywords:** C5 palsy, shoulder traction, cadaveric study, anterior approach, extraforaminal, extradural, foraminal stenosis, central stenosis

## Abstract

*Background and Objectives*: Many risk factors for postoperative C5 palsy (PC5P) have been reported regarding a “cord shift” after a posterior approach. However, there are few reports about shoulder traction as a possible risk factor of anterior cervical surgery. Therefore, we assessed the stretched nerve roots when shoulder traction was applied on cadavers. *Materials and Methods*: Eight cadavers were employed in this study, available based on age and the presence of foramen stenosis. After dissecting the sternocleidomastoid muscle of the cadaver, the shoulder joint was pulled with a force of 2, 5, 8, 10, 15, and 20 kg. Then, the stretched length of the fifth nerve root was measured in the extra-foraminal zone. In addition, the same measurement was performed after cutting the carotid artery to accurately identify the nerve root’s origin. After an additional dissection was performed so that the superior trunk of the brachial plexus could be seen, the stretched length of the fifth and sixth nerve roots was measured again. *Results*: Throughout the entire experiment, the fifth nerve root stretched out for an average of 1.94 mm at 8 kg and an average of 5.03 mm at a maximum force of 20 kg. In three experiments, the elongated lengths of the C5 nerve root at 8 kg and 20 kg were 1.69/4.38 mm, 2.13/5.00 mm, and 0.75/5.31 mm, respectively, and in the third experiment, the elongated length of the C6 nerve root was 1.88/5.44 mm. *Conclusions*: Although this was a cadaveric experiment, it suggests that shoulder traction could be the risk factors for PC5P after anterior cervical surgery. In addition, for patients with foraminal stenosis and central stenosis, the risk would be higher. Therefore, the surgeon should be aware of this, and the patient would need sufficient explanation.

## 1. Introduction

Postoperative C5 palsy (PC5P) is a well-known complication after cervical spine surgery, with an incidence of 6.3% [1]. The difference in incidence depends on the approach and surgical procedures [2,3,4,5]. When an isolated anterior approach was taken, the rate of palsy was 4.3%, while the rate of palsy was 10.9% with an isolated dorsal approach and 11.1% with an antero-posterior procedure. C5 palsy occurs due to a “cord shift” after dorsal decompression, as the C5 nerve root becomes more tethered compared to other cervical roots [3,6,7]. Therefore, procedures such as prophylactic foraminotomy and skip laminectomy/laminoplasty have been attempted in order to reduce tethering of the nerve root due to cord shift [8,9,10,11].

The “cord shift” described above is common in posterior surgery, but PCP5 may also occur after an anterior cervical operation. Therefore, studies to identify other causes have been performed and published [3,7,8,12]. Among them, excessive shoulder traction for fluoroscopic imaging of the lower cervical spine was identified as a risk factor for PC5P [12,13,14,15]. However, previous anatomical studies have only suggested the possibility that the nerve root may be stretched when the shoulder joint undergoes traction [5,12,16]. In addition, since no studies have been performed in an actual surgical setting, it remains unclear whether shoulder traction correlates with clinical features.

In this study, we observed stretching of the fifth cervical root in a cadaver when shoulder traction was applied in an anterior surgical setting. Therefore, our goal is to evaluate the possibility of shoulder traction causing PC5P in anterior cervical surgery.

## 2. Materials and Methods

Permission to perform a cadaver study was granted by the Institutional Review Board of the Yonsei University College of Medicine, Seoul, Republic of Korea (2021-1893-001).

Eight formalin-fixed cadavers provided by our institution for the training of residents were selected. The mean age was 71.75 years (range: 67–77), and all patients were male.

**Figure 1:**
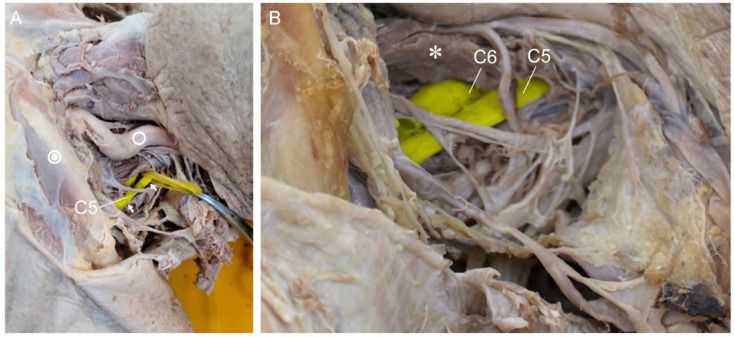
(**A**) Unadulterated photo after the first dissection. The rubber indicates the C5 nerve root. The single circle indicates the carotid artery, and the double circle is the clavicle. The white arrows indicate the proximal and distal segments of the C5 nerve root. (**B**) Lateral view of the more fully dissected left side of the neck. The asterisk indicates the anterior scalene muscle. C5 and C6 nerve roots emerging from this muscle are shown.

**Figure 2:**
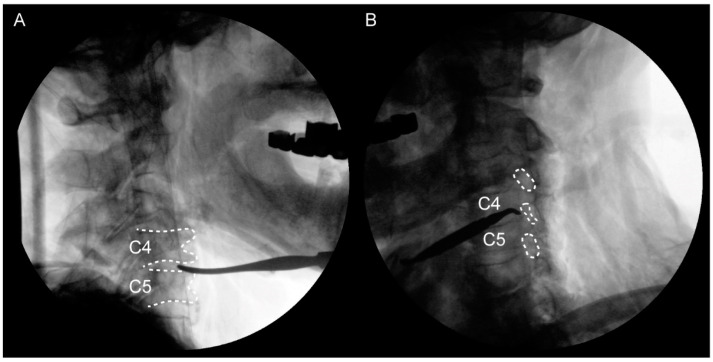
(**A**) Lateral view to confirm the C4–5 disc. (**B**) Oblique view to confirm the C4–5 foramen. The C4–5 foramen is narrower compared to the adjacent level.

The selected cadavers were confirmed to present foraminal stenosis upon oblique viewing by mobile C-arm with 12-inch image intensifier (Ziehm Vision; Ziehm Imaging GmbH, Orlando, FL, USA). This condition could increase the likelihood of C4-5 being pinched in the extradural and extraforaminal zone. Therefore, PC5P might be induced by pre-existing C4–5 foraminal stenosis. In addition, this would make it easier to observe stretching of the nerve root [4,8]. Dissection was performed on the left side of the neck of each cadaver. First, using the Smith–Robinson approach [17], we identified the anterior vertebral body. Sternocleidomastoid muscles (SCMs) were cut at the proximal site, so that the C5 and C6 roots from the anterior scalene could be seen. The distal part was dissected up to the supraclavicular area (to the brachial plexus’ superior trunk) while preserving other structures (Figure 1). To confirm the C5 and C6 roots, C-arm was used to locate the C4–5 and C5–6 foramen (Figure 2). The dissection was performed by two orthopedic surgeons, with a main operator with over 20 years of experience in thousands of cases of anterior cervical spine surgery and an assistant with 2 years of experience in hundreds of cases.

**Figure 3:**
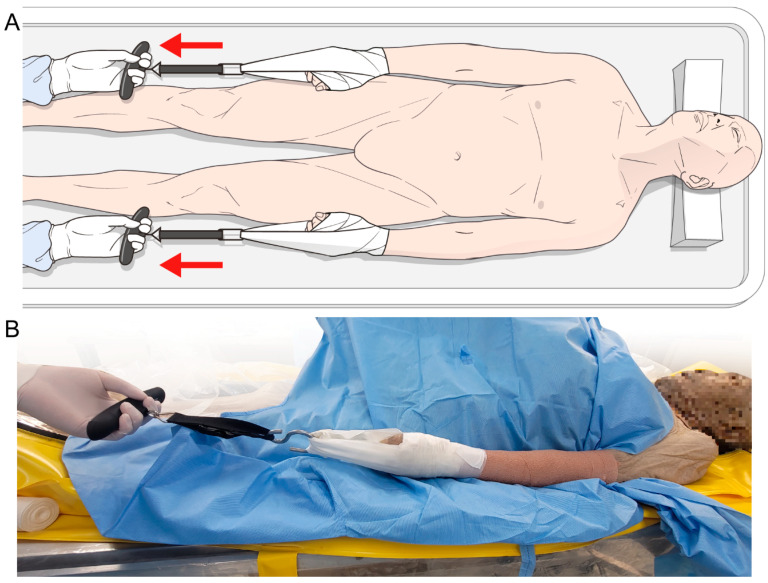
(**A**,**B**) Experimental setting after applying Buck’s traction. The left forearm of the cadaver was pulled horizontal to the floor and upper extremity.

In the supine position, we applied Buck’s traction on the cadaver’s left forearm and connected a force-gauge instrument (TS-50K, TENKYO, Shanghai, China) to the traction. One examiner pulled the device (on the cadaver’s left side) horizontal to the floor and upper extremity. A simultaneous counter force was applied to the foot. The other examiner pulled the right forearm with a similar counter force to obtain the same height of both shoulders (similar to shoulder traction during anterior cervical surgery) (Figure 3).

In the first experiment, the most proximal part of the C5 nerve root, originating from the anterior scalene, was marked 2 cm from the origin. The distance of the root extension was measured by pulling it with a force of 2, 5, 8, 10, 15, or 20 kg.

This traction force was determined using pre-investigated values measured from anesthetized patients who underwent anterior surgery. These values were previously investigated by our institute outside of the current experiment. The average traction force weight was 12.25 kg (8.5–14 kg) for male patients and 9.47 kg (8–13 kg) for female patients. Therefore, a traction force weight of 8, 10, or 15 kg was included. Moreover, to assess the minimum traction force of the elongation, 2 and 5 kg, both lower than 8 kg, were included. Considering the cadaver’s rigidity compared to actual anesthetized patients, a maximum traction force of 20 kg was also applied.

Simultaneously, we took a C-arm image to visualize the lower cervical vertebra in the image when each force was applied. To better visualize the C5 root origin, the carotid artery was cut, and the same estimation was performed (second experiment).

We next dissected the anterior scalene muscles proximally, which allowed us to visualize the origins of the C5 and C6 nerve roots from the foramen. More distal dissection was performed to observe the superior trunk of the brachial plexus where the C5 and C6 roots join distally. The length of C5 and C6 nerve root extensions was measured due to sufficient visualization of the C5 and C6 nerve roots (third experiment). In this setting, for comfortable visualization, distal points of the C5 and C6 nerve roots were marked 2.5 cm from the proximal point, the lower bony margin of the foramen.

In all experiments, a sterile skin marker was used for marking, and a surgical ruler was used to estimate the extended nerve root length. When the ruler moved due to the traction force, a third examiner relocated the proximal point along the nerve root axis and the previously marked proximal point using mosquito forceps.

In this study, SPSS 23.0 (SPSS Inc., Chicago, IL, USA) was used for statistical analysis. A Kruskal–Wallis test was performed, and the criterion for statistical significance was set to *p* < 0.05.

## 3. Results

In all experiments, the length of the nerve root did not extend at a force of 2 kg, but an increase in nerve root length was observed after applying a force of 5 kg (Table 1).

In the first experiment, the force led to an average increase of 0.25 ± 0.43 mm at 5 kg and 4.38 ± 0.48 mm at 20 kg. In the second experiment, conducted after cutting the carotid artery, the stretched length increased from 0.69 ± 0.75 mm at 5 kg to 5.00 ± 1.00 mm at 20 kg, compared to the first experiment (Table 2).

**Figure 4:**
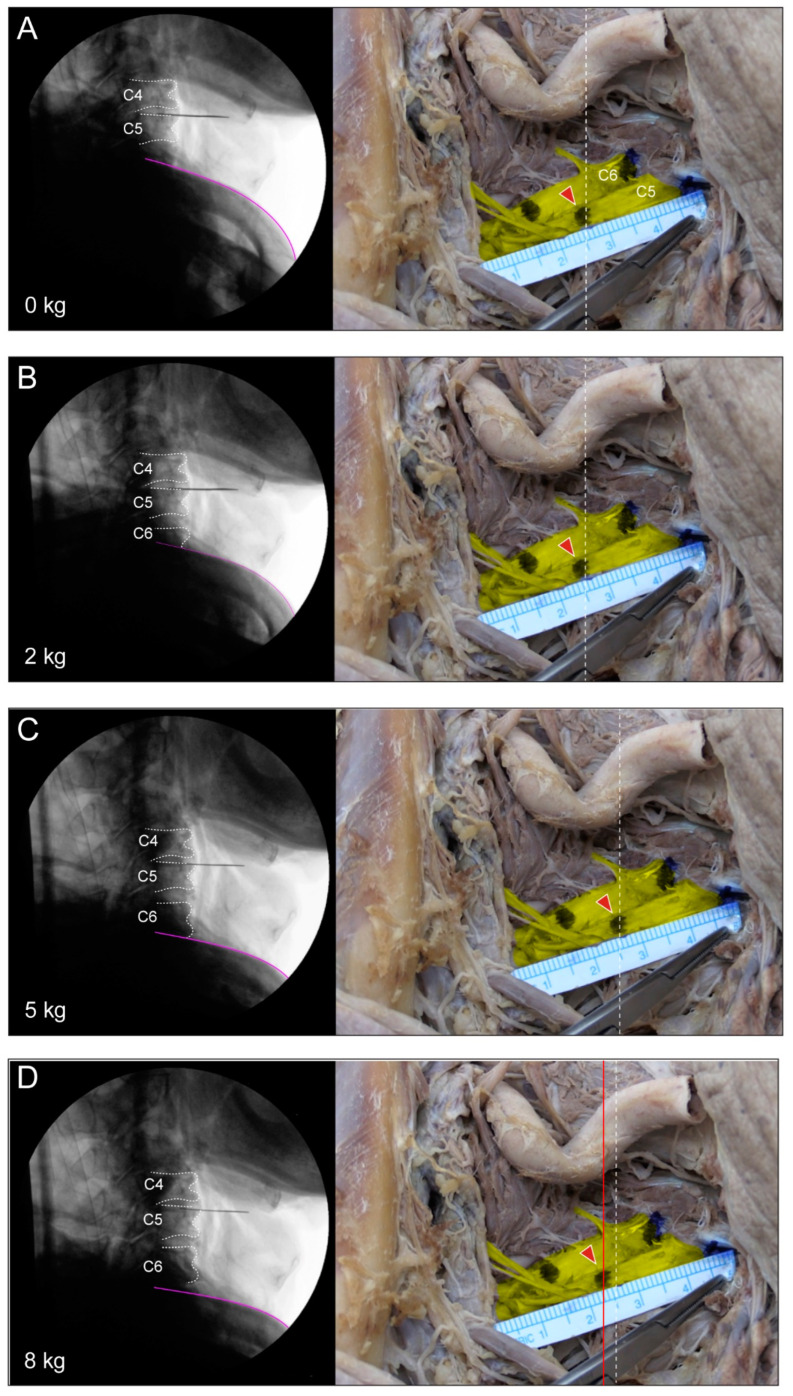

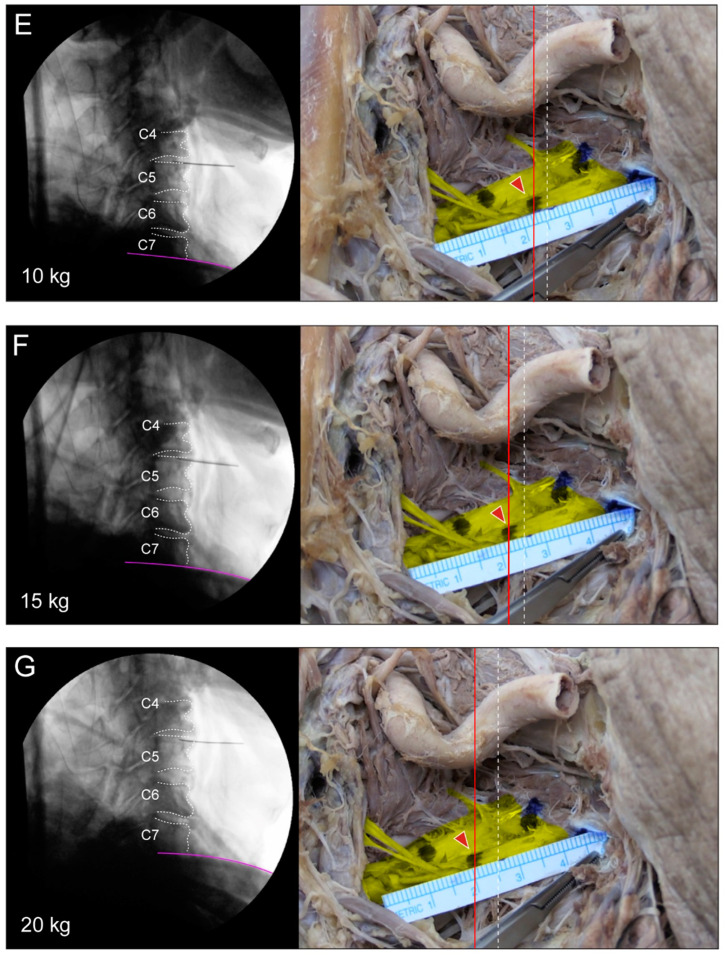
(**A**–**G**) Fluoroscopic lateral view and unadulterated findings of a stretched root at each traction force. Indication of lateral view located in the C4–5 intervertebral space and the inferior purple line indicates the upper margin of the clavicle. Each traction force is 0, 2, 5, 12, 15, and 20 kg. As the traction force increases, the visible range of the lower cervical vertebrae increases in the lateral image, and, finally, the inferior margin of the C7 endplate is visible at a force of 20 kg. The vertical dotted line (white) on the right unadulterated photo shows a point 2.5 cm away from the extraforaminal origin. How much the root stretches as more traction force is applied can be seen by the red arrow with a vertical line (red) and the point marked on the nerve root moving away from the dotted line.

In the third experiment, after further dissection, the mean length of C5 increased from 0.75 mm to 5.31 mm at 5 kg, and that of C6 increased from 0.5 mm to 5.44 mm at 5 kg. The initial extended length measured at 5 kg was slightly shorter for C6, but it was observed that the increase was greater than that for C5 as force was applied (Figure 4, Table 2).

An analysis of the elongation lengths of cervical fifth and sixth nerve roots using a nonparametric test (Kruskal–Wallis test) revealed a significant increase in the average value as the traction force increased (Table 2).

## 4. Discussion

In this study, it was confirmed that the fifth nerve root was stretched in the extraforaminal zone through shoulder joint traction. This is significant in that it suggests one possible cause for PC5P.

The prognosis for PC5P has been reported to be relatively good, with an onset time mostly within a few hours to a few weeks after surgery and spontaneous full recovery achieved within the first 6 months in most cases [3,5,7,8,12]. However, even if the prognosis of PC5P is good, dysfunction due to PC5P may affect postoperative rehabilitation or quality of life and adversely affect the overall prognosis. Therefore, it is important to understand the etiology of PC5P and prevent it in advance.

Risk factors for PC5P include iatrogenic direct nerve root injury or intraoperative traction [18,19]; surgical strategies such as multiple segmental operation with/or posterior approach leading to an extensive spinal cord shift [3,6,20,21]; and shoulder traction for positioning [11,12,13,14].

Among these, surgical strategies are mainly related to the anterior and posterior approaches. The overall incidence of PC5P for anterior approaches is 4.3% and that for dorsal approaches is 10.9%. According to these results, PC5P occurs more in posterior surgery [3,22,23].

This is due to the anatomical features of the C5 nerve root. C5 dorsal rootlets have been found to originate horizontally and are shorter compared to other cervical rootlets [24,25]. Therefore, C5 ventral rootlets become taut and easily injured after dorsal decompression [26,27,28,29,30]. Similarly, PC5P has also been shown to occur after anterior surgery due to the anatomical features described above [20,31]. However, the “cord shift” theory focuses mainly on the posterior approach, and the relationship with anterior surgery has not been explained completely. Therefore, other risk factors of unexpected PC5P should be considered.

Of the risk factors mentioned above, shoulder traction is performed in both anterior and posterior surgeries, but excessive traction is used for lateral imaging of the lower cervical spine, especially in anterior surgery [12,15]. In some studies, upon intraoperative neuromonitoring (ION), the loss of somatosensory evoked potential (SSEP) during anterior surgery returned after releasing the shoulder traction [13,14]. These studies suggest that preoperative shoulder traction may affect PC5P.

Since there are no standardized criteria for traction forces, we had concerns about how much traction force should be applied. Woodroffe applied 10, 20, and 30 lb in five subjects, and then used an MRI to determine the angles between the vertical spinal axis, the C5 nerve root, and the upper trunk [12]. The angle between the C5 root and the upper trunk increased with a higher weight, and it was statistically significant. Therefore, Woodroffe demonstrated that shoulder traction could lead to C5 nerve root tension with subsequent injury and palsy. However, since the standards of 10, 20, and 30 lb used in that study did not reflect the traction force applied in actual operations, we determined the traction forces based on the pre-investigated values from our institute. These values were measured among anesthetized patients who underwent anterior surgery.

Although we have other concerns about nerve dysfunction according to the length of cervical root elongation, there are no precise data on this topic. Some reports of peripheral nerve injuries have shown that the thresholds for nerve rupture and dysfunction are different for each nerve [32,33]. We reviewed studies about the ulnar nerve elongation for reference data on stretched length. These studies showed increased length and strain with elbow movement but did not provide a detailed analysis of the dysfunction associated with an increased length [34,35]. Therefore, it is not possible to know exactly how the increased length in this study affected PC5P.

However, there are some studies that can infer this. When the abduction extension cervical nerve root stress test was performed on radiculopathy patients, pain aggravation or paresthesia occurred, and under the same conditions, the cadaver nerve root was displaced by about 2 to 6 mm [16]. The results of our study showed an increase from about 1.94 to 3.81 mm under the 8 and 15 kg traction forces, which are similar to the traction forces applied during actual surgery. Therefore, we suggest that the stretched nerve root observed in our study could cause neuropathic symptoms. In addition, since this tension is maintained during surgery, patients may progress to PC5P.

In another cadaveric study, the intradural length during shoulder traction was observed after dissecting the dural sac, and the study concluded that shoulder depression could be a risk factor for PC5P [5]. It also reported that intradural movement at the “gutter level” (the transition from inside the foramen to outside the foramen) was much more dramatic. However, that study observed the entire rootlet length regardless of the gutter level. Therefore, to address this limitation, we observed that the cervical nerve root movement grossly passed by the gutter level.

**Figure 5:**
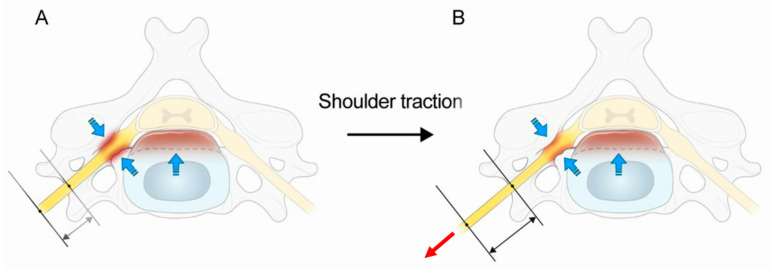
Schematic diagram of the stretched nerve root in the extradural and extraforaminal zone. (**A**) Before shoulder traction, both foraminal stenosis and central stenosis (blue arrows) are shown on the left side of the picture. (**B**) After shoulder traction, only the nerve root in the extradural and extraforaminal zone is stretched because of a pinched “gutter level” (the transition from inside the foramen to outside the foramen) due to foraminal stenosis and cord compression. The blue arrow indicates nerve compression caused by central stenosis and foraminal stenosis, and the red arrow shows the direction in which the nerve root is stretched due to shoulder traction.

Moreover, it has been reported that the main etiology of PC5P is impairment of the C5 nerve root induced by existing C4–5 foraminal stenosis [36]. Therefore, we propose that the nerve root is more easily pinched in the gutter level if there is foraminal stenosis of C4–5. Also, most patients who undergo surgery present myeloradiculopathy, and they have both foraminal stenosis and central stenosis. So, the nerve root is more pinched at the gutter level, since the space of intradural rootlets is reduced. Therefore, the C5 nerve root elongates more in the extraforaminal zone (passed by the gutter level), and this leads to PC5P (Figure 5). So, we recommend that patients with foraminal stenosis and central stenosis are treated more gently and carefully during shoulder traction.

There were some limitations in this study. The C6 extended length measured during the second experiment was the same as the one for the C5 nerve root. This information can be used for a subsequent comparative study in the future, but, for now, there are not enough data to explain its significance. Secondly, this study was limited in that it did not have a control group without foraminal stenosis at C4–5 for comparison. Also, our total of only eight cadavers is not enough to explain the effects of shoulder traction. Therefore, a serial cadaver study with a control group is needed in the future. In addition, we conducted experiments by cutting and dissecting structures such as soft tissues, carotid arteries, and the SCM, so the results may differ from those of actual patients. Thus, further studies are necessary to address and overcome the limitations mentioned above.

In this study, it was observed that shoulder traction could lengthen the cervical nerve roots in formalin-fixed cadavers. However, because the moduli of elasticity and stiffness of formalin-fixed cadavers are higher than those of fresh and fresh/frozen cadavers, these biomechanical differences pose limitations [37]. Nevertheless, these results still hold significant value. In actual patients, because the stiffness and elasticity will be less, the nerve root will be more likely to be stretched, ultimately suggesting a higher likelihood of PC5P.

Additionally, the evaluation of the effect of shoulder traction on the nerve root may be further elucidated in prospective studies using high-field magnetic resonance imaging systems, particularly those which may elucidate the effect of traction on diffusion within the nerve root, which would be valuable to study in combination with these cadaver studies [38].

## 5. Conclusions

The risk factors for PC5P are variable. Our study focused on the effects of shoulder traction performed during preoperative positioning. In the extraforaminal zone, the cadaver’s fifth and sixth cervical nerve roots were both stretched from about 2 mm to 4 mm (mean 1.94 mm, 3.81 mm) when forces applied during actual surgery were used, suggesting that the traction force applied during actual surgery could cause PC5P. In addition, if patients present with foraminal stenosis and central stenosis, this risk will be higher.

Although shoulder traction is necessary for the fluoroscopic imaging of the lower cervical spine, as well as easier approach and wound closure, the surgeon should be aware of the related risks and explain them to the patient.

## Figures and Tables

**Table 1:** Increased lengths (mm) for shoulder traction forces in eight cadavers.

			Increased Length per Each Traction Force (mm)					
Cadaver (Sex/Age)	Nerve Root	Experiment	2 kg	5 kg	8 kg	10 kg	15 kg	20 kg
A (72/M)	C5	1 *	0	0	2	3	4	5
		2 **	0	0	2	3	6.5	7
		3 ***	0	0	2.5	3	4	6
	C6		0	0	1	4	4	5
B (68/M)	C5	1	0	1	2	2	3	4
		2	0	2	3	3	3	4
		3	0	1	1	2	2.5	3
	C6		0	0	2	2	2	5
C (70/M)	C5	1	0	0	1	2	3	4
		2	0	0	2	2	3	4
		3	0	0	2	3	3	5
	C6		0	0	1.5	2	3.5	5
D (73/M)	C5	1	0	1	2	2	4	5
		2	0	1.5	2	3	3	5
		3	0	1	2.5	2.5	4	6
	C6		0	1	2	2.5	4	5.5
E (74/M)	C5	1	0	0	1	2	3.5	4
		2	0	0	1	2	4	5
		3	0	1	1.5	2	4	5.5
	C6		0	1	1.5	3	4	6
F (67/M)	C5	1	0	0	1.5	2	4	5
		2	0	1	2	2	6	6
		3	0	1	2	2.5	5	7
	C6		0	1	2	2.5	4	6
G (73/M)	C5	1	0	0	2	2.5	3	4
		2	0	0	2	3	4	4
		3	0	0	3	3	4	5
	C6		0	0	3	3.5	4.5	6
H (77/M)	C5	1	0	0	2	2.5	3	4
		2	0	1	3	3	4	5
		3	0	2	2	3	4.5	5
	C6		0	1	2	3	4	5
		Mean length	0.00	0.55	1.94	2.58	3.81	5.03

* First experiment, after initial dissection of the C5 nerve root. ** Second experiment, after cutting the carotid artery (to better visualize the C5 root’s origin). *** Third experiment, dissection from the origin of the extraforaminal zone to the location where the superior trunk is visible (C5 and C6 exposed state).

**Table 2:** Mean increased lengths (mm) for shoulder traction forces in eight cadavers.

		Mean Increased Length per Each Traction Force (mm)						
Experiment	Nerve Root	2 kg	5 kg	8 kg	10 kg	15 kg	20 kg	*p*-Value
1 *	C5	0.00	0.25 ± 0.43	1.69 ± 0.43	2.25 ± 0.35	3.44 ± 0.46	4.38 ± 0.48	<0.001
2 **	C5	0.00	0.69 ± 0.75	2.13 ± 0.60	2.63 ± 0.48	4.19 ± 1.27	5.00 ± 1.00	<0.001
3 ***	C5	0.00	0.75 ± 0.66	2.06 ± 0.58	2.63 ± 0.41	3.88 ± 0.74	5.31 ± 1.09	<0.001
	C6	0.00	0.50 ± 0.50	1.88 ± 0.54	2.81 ± 0.66	3.75 ± 0.71	5.44 ± 0.46	<0.001
Mean		0.00	0.55 ± 0.43	1.94 ± 0.42	2.58 ± 0.44	3.81 ± 0.44	5.03 ± 0.49	<0.001

Length is described as “mean ± standard deviation”. * First experiment, after the initial dissection of the C5 nerve root. ** Second experiment, after cutting the carotid artery (to better visualize the C5 root’s origin). *** Third experiment, dissection from the origin of the extraforaminal zone to the location where the superior trunk is visible (C5 and C6 exposed state). The *p*-value was obtained through a Kruskal–Wallis test, with *p* < 0.05 indicating statistical significance.

## Data Availability

Data are contained within the article.

## References

[1] Wang T., Wang H., Liu S., Ding W.Y. (2017). Incidence of C5 nerve root palsy after cervical surgery: A meta-analysis for last decade. Medicine.

[2] Thompson S.E., Smith Z.A., Hsu W.K., Nassr A., Mroz T.E., Fish D.E., Wang J.C., Fehlings M.G., Tannoury C.A., Tannoury T. (2017). C5 palsy after cervical spine surgery: A multicenter retrospective review of 59 cases. Glob. Spine J..

[3] Krätzig T., Mohme M., Mende K.C., Eicker S.O., Floeth F.W. (2017). Impact of the surgical strategy on the incidence of C5 nerve root palsy in decompressive cervical surgery. PLoS ONE.

[4] Alonso F., Voin V., Iwanaga J., Hanscom D., Chapman J.R., Oskouian R.J., Loukas M., Tubbs R.S. (2018). Potential mechanism for some postoperative C5 palsies. Spine.

[5] Lee D.H., Lee H.R., Riew K.D. (2024). An Algorithmic Roadmap for the Surgical Management of Degenerative Cervical Myelopathy: A Narrative Review. Asian Spine J..

[6] Yang L., Gu Y., Shi J., Gao R., Liu Y., Li J., Yuan W. (2013). Modified plate-only open-door laminoplasty versus laminectomy and fusion for the treatment of cervical stenotic myelopathy. Orthopedics.

[7] Baba S., Ikuta K., Ikeuchi H., Shiraki M., Komiya N., Kitamura T., Senba H., Shidahara S. (2016). Risk factor analysis for C5 palsy after double-door laminoplasty for cervical spondylotic myelopathy. Asian Spine J..

[8] Choi S.H., Kang C.-N. (2020). Degenerative cervical myelopathy: Pathophysiology and current treatment strategies. Asian Spine J..

[9] Katsumi K., Yamazaki A., Watanabe K., Ohashi M., Shoji H. (2012). Can Prophylactic Bilateral C4/C5 Foraminotomy Prevent Postoperative C5 Palsy after Open-Door Laminoplasty?: A Prospective Study. Spine.

[10] Komagata M., Nishiyama M., Endo K., Ikegami H., Tanaka S., Imakiire A. (2004). Prophylaxis of C5 palsy after cervical expansive laminoplasty by bilateral partial foraminotomy. Spine J..

[11] Nori S., Shiraishi T., Aoyama R. (2020). Comparison between muscle-preserving selective laminectomy and laminoplasty for multilevel cervical spondylotic myelopathy. Ann. Transl. Med..

[12] Woodroffe R.W., Helland L.C., Bryant A., Nourski K.V., Yamaguchi S., Close L., Noeller J., Teferi N., Maley J.E., Hitchon P.W. (2020). Intraoperative shoulder traction as cause of C5 palsy: Magnetic resonance imaging study. World Neurosurg..

[13] Roh M.S., Wilson-Holden T.J., Padberg A.M., Park J.-B., Riew K.D. (2007). The utility of somatosensory evoked potential monitoring during cervical spine surgery: How often does it prompt intervention and affect outcome?. Asian Spine J..

[14] Yoshihara H., Pivec R., Naam A. (2018). Positioning-related neuromonitoring change during anterior cervical discectomy and fusion. World Neurosurg..

[15] Truong V.T., Al-Shakfa F., Boubez G., Shedid D., Yuh S.-J., Wang Z. (2020). Enhanced Visualization of the Cervical Vertebra during Intraoperative Fluoroscopy Using a Shoulder Traction Device. Asian Spine J..

[16] Farshad M., Min K. (2013). Abduction extension cervical nerve root stress test: Anatomical basis and clinical relevance. Eur. Spine J..

[17] Baker A.D. (2014). The treatment of certain cervical-spine disorders by anterior removal of the intervertebral disc and interbody fusion. Classic Papers in Orthopaedics.

[18] Hirabayashi K., Satomi K. (1988). Operative procedure and results of expansive open-door laminoplasty. Spine.

[19] Liu T., Zou W., Han Y., Wang Y. (2010). Correlative study of nerve root palsy and cervical posterior decompression laminectomy and internal fixation. Orthopedics.

[20] Park S., Lee D.H., Lee C.S., Hwang C.J., Yang J.J., Cho J.H. (2023). Anterior Decompression and Fusion for the Treatment of Cervical Myelopathy Caused by Ossification of the Posterior Longitudinal Ligament: A Narrative Review. Asian Spine J..

[21] Choi J.H., Birring P.S., Lee J., Hashmi S.Z., Bhatia N.N., Lee Y.P. (2024). A Comparison of Short-Term Outcomes after Surgical Treatment of Multilevel Degenerative Cervical Myelopathy in the Geriatric Patient Population: An Analysis of the National Surgical Quality Improvement Program Database 2010–2020. Asian Spine J..

[22] Eun D.C., Suguitan A.A., Suk K.S., Kim H.S., Kwon J.W., Moon S.H., Lee Y.H., Lee B.H. (2022). Variation in Prevertebral Soft Tissue Swelling after Staged Combined Multilevel Anterior-Posterior Complex Cervical Spine Surgery: Anterior Then Posterior (AP) versus Posterior Then Anterior-Posterior (PAP) Surgery. J. Clin. Med..

[23] Kwon J.W., Bang S.H., Park T.H., Lee S.J., Lee H.M., Lee S.B., Lee B.H., Moon S.H. (2020). Biomechanical comparison of cervical discectomy/fusion model using allograft spacers between anterior and posterior fixation methods (lateral mass and pedicle screw). Clin. Biomech..

[24] Alleyne Jr C.H., Cawley C.M., Barrow D.L., Bonner G.D. (1998). Microsurgical anatomy of the dorsal cervical nerve roots and the cervical dorsal root ganglion/ventral root complexes. Surg. Neurol..

[25] Shinomiya K., Okawa A., Nakao K., Mochida K., Haro H., Sato T., Heima S. (1994). Morphology of C5 ventral nerve rootlets as part of dissociated motor loss of deltoid muscle. Spine.

[26] Lee Y.H., Abdou M., Kwon J.W., Suk K.S., Moon S.H., Won Y.G., Lee T.J., Lee B.H. (2023). Posterior Preventive Foraminotomy before Laminectomy Combined with Pedicle Screw Fixation May Decrease the Incidence of C5 Palsy in Complex Cervical Spine Surgery in Patients with Severe Myeloradiculopathy. J. Clin. Med..

[27] Kwon J.W., Arreza E.O., Suguitan A.A., Lee S.B., Sung S., Park Y., Ha J.W., Kim T.H., Moon S.H., Lee B.H. (2022). Medial Pedicle Pivot Point Using Preoperative Computed Tomography Morphometric Measurements for Cervical Pedicle Screw Insertion: A Novel Technique and Case Series. J. Clin. Med..

[28] Kim S.H., Kim J.H., Kwon J.W., Kim H.S., Moon S.H., Suk K.S., Lee B.H. (2023). Assessment of Biomechanical Advantages in Combined Anterior-Posterior Cervical Spine Surgery by Radiological Outcomes: Pedicle Screws over Lateral Mass Screws. J. Clin. Med..

[29] Pennington Z., Lubelski D., Westbroek E.M., Cottrill E., Ehresman J., Goodwin M.L., Lo S.F., Witham T.F., Theodore N., Bydon A. (2020). Spinal cord float back is not an independent predictor of postoperative C5 palsy in patients undergoing posterior cervical decompression. Spine J..

[30] Yokota A., Fujishiro T., Usami Y., Neo M. (2022). An Experimental Rat Model of C5 Palsy Following Posterior Decompression Surgery of the Cervical Spine. Spine.

[31] Kim S., Lee S.-H., Kim E.-S., Eoh W. (2014). Clinical and radiographic analysis of c5 palsy after anterior cervical decompression and fusion for cervical degenerative disease. Clin. Spine Surg..

[32] Mahan M.A. (2019). Nerve stretching: A history of tension. J. Neurosurg..

[33] Mahan M.A., Yeoh S., Monson K., Light A. (2019). Rapid stretch injury to peripheral nerves: Biomechanical results. Neurosurgery.

[34] Mahan M.A., Vaz K.M., Weingarten D., Brown J.M., Shah S.B. (2015). Altered ulnar nerve kinematic behavior in a cadaver model of entrapment. Neurosurgery.

[35] Nagashima M., Omokawa S., Nakanishi Y., Mahakkanukrauh P., Hasegawa H., Tanaka Y. (2021). A Cadaveric Study of Ulnar Nerve Movement and Strain around the Elbow Joint. Appl. Sci..

[36] Katsumi K., Yamazaki A., Watanabe K., Ohashi M., Shoji H. (2013). Analysis of C5 palsy after cervical open-door laminoplasty: Relationship between C5 palsy and foraminal stenosis. Clin. Spine Surg..

[37] Hohmann E., Keough N., Glatt V., Tetsworth K., Putz R., Imhoff A. (2019). The mechanical properties of fresh versus fresh/frozen and preserved (Thiel and Formalin) long head of biceps tendons: A cadaveric investigation. Ann. Anat..

[38] Pušnik L., Serša I., Umek N., Cvetko E., Snoj Ž. (2023). Correlation between diffusion tensor indices and fascicular morphometric parameters of peripheral nerve. Front. Physiol..

